# Sodium Levels Predict Disability at Discharge in Guillain-Barré Syndrome: A Retrospective Cohort Study

**DOI:** 10.3389/fneur.2021.729252

**Published:** 2021-09-07

**Authors:** Delia Gagliardi, Irene Faravelli, Manuel Alfredo Podestà, Roberta Brusa, Eleonora Mauri, Domenica Saccomanno, Alessio Di Fonzo, Sara Bonato, Elio Scarpini, Nereo Bresolin, Giacomo Pietro Comi, Stefania Corti

**Affiliations:** ^1^Neuroscience Section, Dino Ferrari Centre, Department of Pathophysiology and Transplantation, University of Milan, Milan, Italy; ^2^Foundation IRCCS Ca' Granda Ospedale Maggiore Policlinico, Neurology Unit, Milan, Italy; ^3^Renal Division, Azienda Socio Sanitaria Territoriale (ASST) Santi Paolo e Carlo, Department of Health Sciences, University of Milan, Milan, Italy; ^4^Stroke Unit, Foundation IRCCS Ca' Granda, Ospedale Maggiore Policlinico, Milan, Italy; ^5^Foundation IRCCS Ca' Granda Ospedale Maggiore Policlinico, Neurodegenerative Unitá Operativa Semplice Dipartimentale (UOSD), Milan, Italy; ^6^Department of Biomedical, Surgical, and Dental Sciences, Dino Ferrari Center, University of Milan, Milan, Italy; ^7^Foundation IRCCS Ca' Granda Ospedale Maggiore Policlinico, Neurodegenerative Unitá Operativa Semplice Dipartimentale (UOSD), Milan, Italy

**Keywords:** hyponatremia, Guillain-Barré, polyradiculopathy, disability, intravenous immunoglobulin

## Abstract

Guillain-Barré syndrome (GBS) is an inflammatory polyradiculopathy with potentially severe complications. Clinical tools for risk stratification have been developed, but no definitive prognostic biomarker has been reported. Hyponatremia is frequent in GBS patients, but the impact of serum sodium levels on clinical outcomes is still ill-defined. In this retrospective cohort study, we included all adult patients diagnosed with GBS spectrum disorders at our center from January 2010 to July 2020. Disability at discharge was assessed with the GBS Disability Score (GDS), and all clinical and laboratory data was retrieved from medical charts. Thirty (58.8%) of the 51 subjects included in the study were discharged with severe residual disability (GDS ≥ 3). After accounting for relevant confounders, the odds of experiencing severe disability decreased by 27% (*p* = 0.027) for each unitary increase in serum sodium concentration. Thirteen (25.5%) patients were diagnosed with mild to moderate hyponatremia; the use of intravenous immune globulin (IVIG) independently increased the odds of developing hyponatremia. In conclusion, we found a significant, independent association between baseline serum sodium levels and severe disability at discharge in GBS patients. In our cohort, hyponatremia was more frequently observed after treatment with IVIG, suggesting dilutional pseudohyponatremia as a probable cause.

## Introduction

Guillain-Barré syndrome (GBS) is an inflammatory polyradiculopathy with a worldwide incidence of 100,000 cases per year, which can lead to permanent severe disability in a significant fraction of patients (1). Several clinical tools for risk stratification have been developed to tailor therapeutic strategies (2), but no reliable prognostic biomarker has been reported to date.

Hyponatremia is one of the most frequent electrolyte abnormalities observed in hospitalized and critically-ill patients (3, 4), and sodium levels at admission have been found to predict mortality in various patient populations (5, 6). The occurrence of hyponatremia in GBS patients has been first reported decades ago (7, 8), but the impact of serum sodium levels on clinical outcomes is still ill-defined (9, 10).

In the present study, we have assessed sodium levels in a well-characterized monocentric cohort of GBS patients, in order to explore their association with clinical outcomes and potential causative determinants of hyponatremia during hospitalization.

## Methods

### Study Design and Selection Criteria

In this retrospective cohort study, we included all adult patients with GBS spectrum disorders admitted to the Neurology Unit of the IRCCS Fondazione Ca' Granda Ospedale Policlinico di Milano from January 2010 to July 2020. All patients enrolled in the study met diagnostic criteria for GBS or Miller Fisher syndrome (11, 12). Patients with incomplete clinical information from medical records were excluded. The study was conducted in accordance with the Declaration of Helsinki and received approval by the Local Ethics Committee.

### Outcomes and Assessments

The primary aim of the study was to evaluate the impact of baseline sodium levels on disability at discharge. Disability was assessed with the GBS Disability Score (GDS), which ranges from 0 (no limitation) to 6 (death); severe disability was defined by a GDS ≥ 3, i.e., inability to walk without assistance for 10 meters across an open space. The association of demographic features, clinical characteristics and in-patient treatments with the development of hyponatremia (defined as serum sodium levels < 135 mEq/L) during hospitalization was also assessed as a secondary outcome.

Patient demographics, premorbid disability (modified Rankin scale—mRS), past medical history, concomitant medications and neurologic symptoms throughout hospitalization were collected from hospital records. Cerebrospinal fluid protein concentration, serology for anti-ganglioside antibodies and sodium levels on admission and during hospitalization were retrieved from centralized laboratory results. Electrophysiological analysis—electroneurography (ENG) and electromyography (EMG)—were performed by evaluating nerve conduction in at least two motor and two sensory nerves in upper and lower limbs, and demyelinating or axonal GBS was defined according to standard criteria (13). GBS-specific treatment comprised plasma exchange (PLEX) and/or intravenous immunoglobulin (IVIG) infusion, based on clinical judgment.

### Statistical Analyses

Baseline characteristics were analyzed through descriptive statistics, and baseline stratification between patients with high- and low-sodium concentration was performed according to an arbitrarily defined cut-off (median of the normal range, i.e., 140 mEq/L). Between-group comparisons were performed with Mann Whitney or Fisher's exact-test, as appropriate. Differences in paired samples were assessed with the Wilcoxon signed-rank test. Correlation between continuous variables was evaluated with the Spearman's coefficient. Univariable and multivariable binomial logistic regression models were employed to evaluate the association between variables of interest with either the occurrence of severe disability at discharge or that of hyponatremia during hospitalization. Covariate selection for multivariable models was based on clinical relevance. Statistical analyses were performed with R version 4.0.0 (R Foundation for Statistical Computing, Vienna, Austria).

### Data Availability Statement

De-identified participant data used in this work are available upon reasonable request. To gain access, researchers will need to submit a methodologically sound proposal to the corresponding author, sign a data access agreement and obtain the approval of the local ethics committee.

## Results

### Disposition of Subjects and Baseline Characteristics

**Figure 1:**
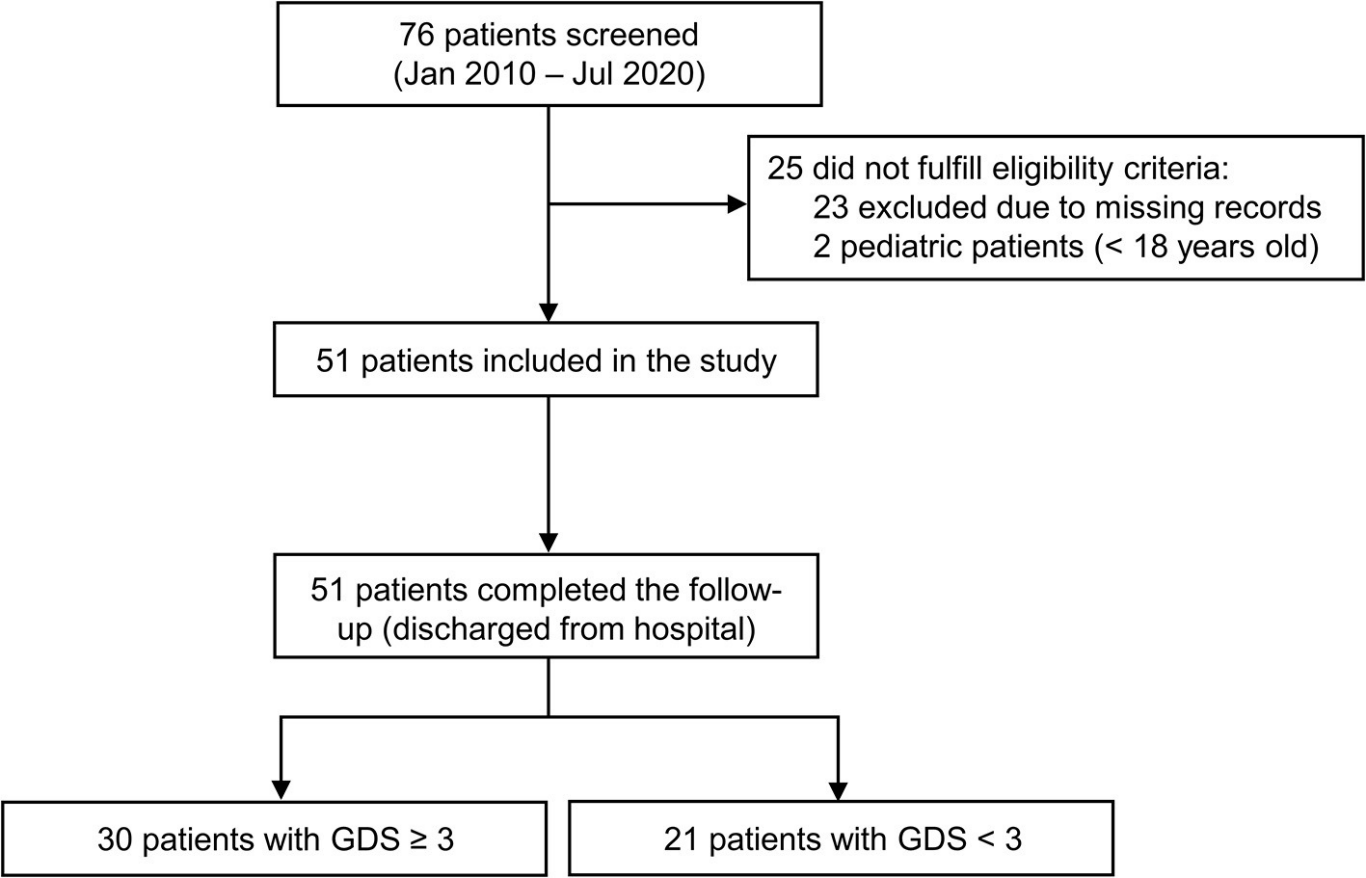
Study flow-chart. All adult patients with GBS spectrum and complete clinical information from medical records were included.

We screened 76 patients admitted to our unit between January 2010 and July 2020, of whom 25 were excluded due to ineligibility (Figure 1). Hence, 51 patients with complete medical records were included in this study. After stratification according to baseline serum sodium levels, demographic and clinical features were balanced between groups (Table 1). Patients with lower serum sodium at baseline (<140 mEq/L) tended to be older than patients with higher sodium values, but the use of medications that could potentially increase the risk of hyponatremia was similar between groups.

**Table 1:** Baseline characteristics of 51 patients diagnosed with GBS at a single Italian institution.

	**Overall (*n* = 51)**	**Baseline Na^**+**^ ≥ 140 mEq/L (*n* = 30)**	**Baseline Na^**+**^ <140 mEq/L (*n* = 21)**	***p*-value**
Age (years)	56.0 (34.0–70.0)	43.5 (29.5–70.0)	66.0 (48.0–72.0)	0.054
Sex (M, %)	30 (58.8%)	20 (66.7%)	10 (47.6%)	0.249
Mod. Rankin scale (score, %)				0.079
0	42 (82.4%)	24 (80.0%)	18 (85.7%)	
1	3 (5.9%)	1 (3.3%)	2 (9.5%)	
2	5 (9.8%)	5 (16.7%)	0 (0.0%)	
3	1 (2.0%)	0 (0.0%)	1 (4.8%)	
Symptom onset (days)	6.0 (3.0–14.5)	9.5 (3.0–15.0)	5.0 (5.0–8.0)	0.265
Previous infection (Yes, %)	35 (68.6%)	23 (76.7%)	12 (57.1%)	0.220
GBS type (MFS, %)	3 (5.9%)	2 (6.7%)	1 (4.8%)	1.000
Involvement (Yes, %)				
Bulbar	20 (39.2%)	11 (36.7%)	9 (42.9%)	0.773
Cranial	20 (39.2%)	12 (40.0%)	8 (38.1%)	1.000
Respiratory	10 (19.6%)	6 (20.0%)	4 (19.0%)	1.000
Motor	44 (86.3%)	25 (83.3%)	19 (90.5%)	0.685
Sensorial	38 (74.5%)	20 (66.7%)	18 (85.7%)	0.193
Pain	22 (43.1%)	12 (40.0%)	10 (47.6%)	0.774
Dysautonomia	21 (41.2%)	13 (43.3%)	8 (38.1%)	0.778
Areflexia	49 (96.1%)	28 (93.3%)	21 (100.0%)	0.506
CSF protein (mg/dL)	82.0 (53.3–113.0)	87.0 (51.0–113.0)	82.0 (59.5–98.0)	0.792
Hematocrit (%)	40.9 (37.0–43.8)	42.0 (37.3–43.9)	38.7 (35.9–43.6)	0.140
Anti-ganglioside Ab (%)				0.278
Positive	32 (62.7%)	22 (73.3%)	10 (47.6%)	
Negative	11 (21.6%)	5 (16.7%)	6 (28.6%)	
NA	8 (15.7%)	3 (10.0%)	5 (23.8%)	
EMG findings (%)				0.762
Axonal	18 (35.3%)	10 (33.3%)	8 (38.1%)	
Demyelinating	30 (58.8%)	19 (63.3%)	11 (52.4%)	
NA	3 (5.9%)	1 (3.3%)	2 (9.5%)	
Neuropathy type (%)				0.854
Motor	30 (58.8%)	19 (63.3%)	11 (52.4%)	
Sensory	1 (2.0%)	1 (3.3%)	0 (0.0%)	
Both	18 (35.3%)	10 (33.3%)	8 (38.1%)	
NA	2 (3.9%)	0 (0.0%)	2 (9.5%)	
Concomitant therapy (Yes, %)				
Thiazide Diuretics	3 (5.9%)	1 (3.3%)	2 (9.5%)	0.561
Antidepressants	8 (15.7%)	5 (16.7%)	3 (14.3%)	1.000
Anticonvulsants	3 (5.9%)	1 (3.3%)	2 (9.5%)	0.561
Antipsychotics	2 (3.9%)	2 (6.7%)	0 (0.0%)	0.506
PPI	10 (19.6%)	5 (16.7%)	5 (23.8%)	0.722
NSAID	3 (5.9%)	2 (6.7%)	1 (4.8%)	1.000

*Variables are reported as median (IQR) or number (percentage), as appropriate. Ab, antibodies; CSF, cerebrospinal fluid; EMG, electromyography; PPI, proton-pump inhibitors; NSAID, non-steroidal anti-inflammatory drugs*.

On admission, 44 (86.3%) subjects experienced motor symptoms, 38 (74.5%) had sensory deficits, and all but two (96.1%) presented with areflexia. Anti-ganglioside antibodies were positive in 32 (62.7%) patients, and acute axonal damage was found at ENG/EMG in 18 (35.3%) of cases. During hospitalization, forty-nine patients received GBS-specific treatment: of these, 23 (46.9%) underwent PLEX, 13 (26.5%) were treated with IVIG and 13 (26.5%) received both.

### Disability at Discharge

After a median hospitalization time of 16 (12–25) days all patients were discharged, 30 (58.8%) of whom had severe residual disability (GDS ≥ 3). Factors associated with significantly reduced odds of severe disability at discharge included higher baseline serum sodium levels [OR (95%CI): 0.77 (0.62–0.96), *p* = 0.021] and lack of acute axonal injury [OR (95%CI): 0.11 (0.02–0.56), *p* = 0.008], whereas the presence of motor symptoms was directly associated with this outcome [OR (95%CI): 11.60 (1.28–105.41), *p* = 0.03].

Notably, after correcting for age and presence of axonal injury, the odds of experiencing severe disability decreased by 27% (*p* = 0.027) for each unitary increase in serum sodium concentration (Table 2). In the same multivariable model, the presence of acute axonal injury was independently associated with higher odds of severe disability at discharge [OR (95%CI): 0.09 (0.01–0.51), *p* = 0.007].

**Table 2:** Association between baseline features and residual disability at discharge.

	**Univariable**		**Multivariable**	
	**OR (95%CI)**	***p*-value**	**OR (95%CI)**	***p*-value**
Age (10 years)	1.26 (0.94–1.67)	0.120	1.1 (0.75–1.61)	0.62
Male sex (ref = F)	1.57 (0.51–4.88)	0.440	-	-
Sodium baseline (mEq/L)	0.77 (0.62–0.96)	0.021	0.73 (0.55–0.96)	0.027
Mod. Rankin scale (score)	1.95 (0.72–5.25)	0.190	-	-
Previous infection (ref = No)	0.35 (0.10–1.31)	0.120	-	-
Miller-Fisher syndrome (ref = GBS)	0.33 (0.03–3.97)	0.380	-	-
Acute axonal injury (ref = Yes)	0.11 (0.02–0.56)	0.008	0.09 (0.01–0.51)	0.007
Involvement (ref = No)				
Bulbar	3.20 (0.93–10.98)	0.060	–	–
Cranial	0.77 (0.25–2.41)	0.660	–	–
Respiratory	3.45 (0.65–18.29)	0.140	–	–
Motor	11.60 (1.28–105.41)	0.030	–	–
Sensorial	0.86 (0.24–3.12)	0.820	–	–
Pain	0.73 (0.24–2.26)	0.590	–	–
Dysautonomia	2.50 (0.76–8.19)	0.130	–	–
Anti-Ganglioside Ab Pos. (ref = Neg)	2.35 (0.53–10.52)	0.260	–	–

*Univariable and multivariable binomial logistic regression models using global disability score ≥ 3 at discharge as the outcome*.

### Serum Sodium Levels During Hospitalization

**Figure 2:**
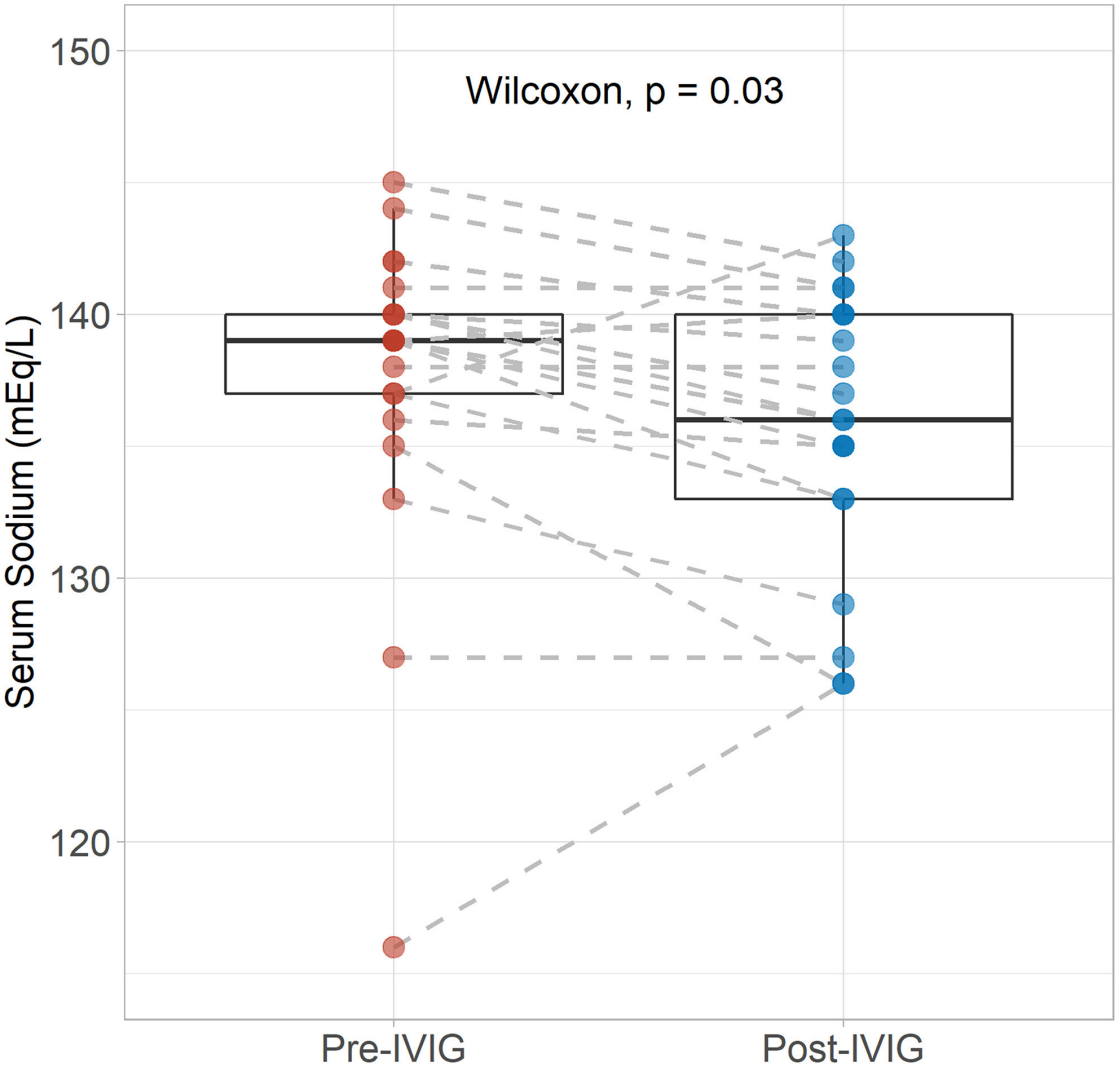
Serum sodium levels before and after completion of treatment with intravenous immunoglobulins. Differences between pre- and post-values in patients receiving IVIG were assessed with the Wilcoxon singed-rank test.

Nadir sodium levels measured throughout hospital stay were directly correlated with baseline sodium concentration (*R* = 0.72, *p* < 0.001), whereas an inverse correlation was found between nadir sodium levels and age (*R* = −0.46, *p* < 0.001). On the other hand, nadir sodium levels did not significantly differ between males and females. Interestingly, nadir serum sodium concentration was significantly lower in patients treated with IVIG compared to patients who did not receive this therapy (*p* < 0.001) (Supplementary Figure 1). Consistently, in these patients, serum sodium levels were significantly reduced after IVIG infusion compared to values recorded before treatment (*p* = 0.03) (Figure 2).

During hospitalization, 13 (25.5%) patients were diagnosed with hyponatremia (nadir serum sodium level < 135 mEq/L), with only one case (2.0%) classified as profound hyponatremia (<125 mEq/L). Univariable and multivariable linear regression analyses were performed to identify factors associated with hyponatremia during hospitalization (Supplementary Table 1). Consistent with results outlined above, after adjusting for age and baseline serum sodium levels, the use of IVIG was independently associated with the development of hyponatremia [OR (95%CI): 20.57 (2.41–175.43), *p* = 0.006].

## Discussion

In this retrospective cohort study, we have found a significant, independent association between baseline serum sodium levels and severe disability at discharge in subjects with GBS spectrum disorders. In these patients, the development of hyponatremia during hospitalization was common, and was more frequently observed after treatment with IVIG.

Previous reports have outlined the existence of a direct association between mortality and hyponatraemia in patients with GBS (10); however, hyponatremia was not a significant predictor of neuromuscular weakness (6). Similarly, a recent prospective study from India has reported an exceptionally high incidence of hyponatremia due to the syndrome of inappropriate secretion of antidiuretic hormone in GBS, which was associated with an increased mortality risk (7). The pathogenesis of hyponatremia in the context of GBS is incompletely understood, but lower baseline sodium levels could represent a marker of a more aggressive disease phenotype in these patients (14).

On the other hand, the development of hyponatremia during hospitalization may be frequently due to treatment with IVIG, which can induce this anomaly through several mechanisms: analyte interference due to the higher protein phase (pseudohyponatremia), hypertonicity resulting from sucrose contained in IVIG preparations, and a true dilutional effect from increased oncotic pressure (15). Our data is consistent with this hypothesis, suggesting that several of the previously described cases of hyponatremia in GBS patients could be due to this mechanism. However, due to the lack of essential diagnostic elements in our study, we cannot rule out other possible causes of hyponatremia, such as SIADH and salt wasting syndrome, which have both been reported in association with GBS (16–18).

Owing to its retrospective design, our study has several limitations, including the lack of information regarding the cause of hyponatremia and the non-standardized therapeutic management in our patient cohort. For these reasons, our findings warrant confirmation in larger longitudinal studies exploring the relevance of sodium levels as a biomarker of GBS severity.

## Data Availability Statement

The original contributions presented in the study are included in the article/Supplementary Material, further inquiries can be directed to the corresponding author.

## Ethics Statement

The studies involving human participants were reviewed and approved by Institutional Review Board of Fondazione IRCCS Ca' Granda Ospedale Maggiore Policlinico. Written informed consent for participation was not required for this study in accordance with the national legislation and the institutional requirements.

## Author Contributions

DG and IF: design and conceptualized study, acquired and interpreted the data, and drafted the manuscript. MP: analyzed and interpreted the data, and drafted the manuscript. RB and EM: acquired and interpreted the data, and drafted the manuscript. DS: acquired the data. AD, SB, ES, NB, GC, and SC: revised the manuscript for intellectual content. All authors contributed to the article and approved the submitted version.

## Funding

This study was supported by Fondazione Regionale per la Ricerca Biomedica (FRRB) Grant An integrated omics approach for patients with rare neurological disorders: toward personalized clinical care and trial readiness (Care4NeuroRare), to SC and Ministery of Health Ricerca Corrente to GC and NB.

## Conflict of Interest

The authors declare that the research was conducted in the absence of any commercial or financial relationships that could be construed as a potential conflict of interest.

## Publisher's Note

All claims expressed in this article are solely those of the authors and do not necessarily represent those of their affiliated organizations, or those of the publisher, the editors and the reviewers. Any product that may be evaluated in this article, or claim that may be made by its manufacturer, is not guaranteed or endorsed by the publisher.

## Supplementary Material

Supplementary Figure 1 Exploratory analyses on the association between nadir sodium levels (mEq/L) during hospitalization and selected clinical features. The correlation between nadir sodium and continuous variables (baseline sodium and age) was summarized with the Spearman coefficient, while differences in nadir sodium levels among the strata of categorical variables (sex and IVIG) were assessed with the Mann-Whitney U-test.

Supplementary Table 1 Association between the occurrence of hyponatremia during hospitalization and clinical features. Univariable and multivariable binomial logistic regression models with hyponatremia (serum sodium < 135 mEq/L) at any time during hospitalization as outcome. ^*^Log-transformed.
