# Supplementary material for: Sodium Levels Predict Disability at Discharge in Guillain-Barré Syndrome: A Retrospective Cohort Study
Source: Front Neurol. 2021 Sep 7;12:729252. doi: 10.3389/fneur.2021.729252 (PMC8453067; doi:10.3389/fneur.2021.729252)
Supplement: Supplementary Figure 1 — Exploratory analyses on the association between nadir sodium levels (mEq/L) during hospitalization and selected clinical features. The correlation between nadir sodium and continuous variables (baseline sodium and age) was summarized with the Spearman coefficient, while differences in nadir sodium levels among the strata of categorical variables (sex and IVIG) were assessed with the Mann-Whitney U-test. [file Table_1.pdf]

SUPPLEMENTARY MATERIAL

Supplementary Figure 1. Exploratory analyses on the association between nadir sodium levels (mEq/L) during hospitalization and selected clinical features. The correlation between nadir sodium and continuous variables (baseline sodium and age) was summarized with the Spearman coefficient, while differences in nadir sodium levels among the strata of categorical variables (sex and IVIG) were assessed with the Mann-Whitney U-test.

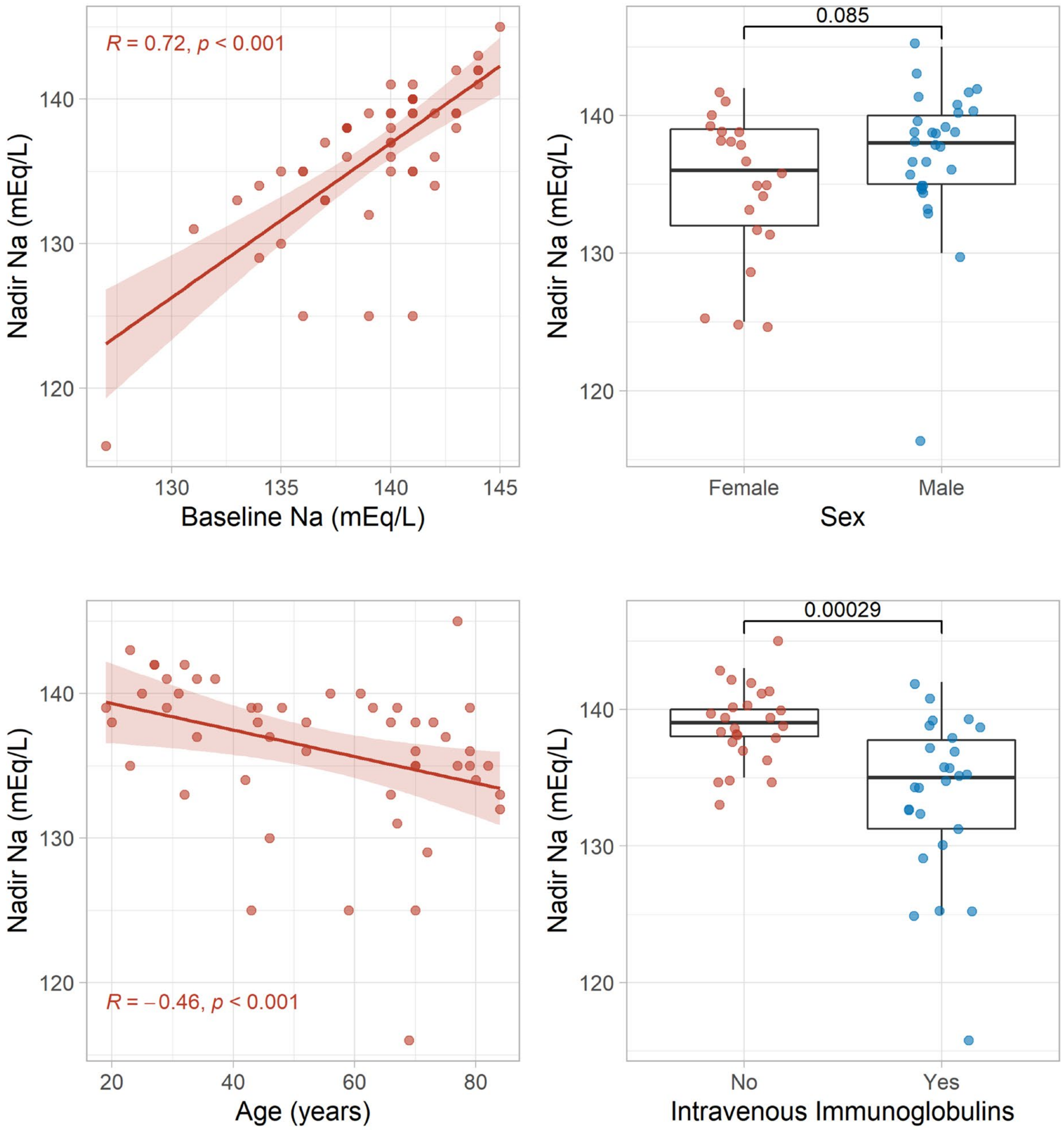

Supplementary Table 1. Association between the occurrence of hyponatremia during hospitalization and clinical features. Univariable and multivariable binomial logistic regression models with hyponatremia (serum sodium < 135 mEq/L) at any time during hospitalization as outcome. *Log-transformed.

	Univariable		Multivariable	
	**OR [95%CI]**	**p-val**	**OR [95%CI]**	**p-val**
**Age** (10 years)	1.37 [0.96-1.94]	0.080	1.23 [0.71-2.16]	0.461
**Male Sex** (ref=F)	0.32 [0.09-1.20]	0.091	-	-
**Sodium Baseline** (mEq/L)	0.60 [0.44-0.82]	0.001	0.52 [0.33-0.83]	0.006
**Mod. Rankin Scale** (score)	1.65 [0.75-3.62]	0.213	-	-
**Symptom Onset** (days)*	1.29 [0.69-2.43]	0.428	-	-
**Previous Infection** (ref=No)	0.65 [0.17-2.44]	0.525	-	-
**Involvement** (ref=No)				
Bulbar	2.24 [0.62-8.07]	0.216	-	-
Cranial	0.96 [0.26-3.49]	0.949	-	-
Respiratory	2.37 [0.55-10.26]	0.248	-	-
Motor	2.25 [0.24-20.69]	0.474	-	-
Sensorial	5.54 [0.64-47.62]	0.119	-	-
Pain	1.18 [0.33-4.18]	0.799	-	-
Dysautonomia	3.08 [0.84-11.32]	0.091	-	-
**Anti-Ganglioside Ab Pos.** (ref=Neg)	1.63 [0.33-8.02]	0.551	-	-
**Demyelinating Neuropathy** (ref=Axonal)	0.31 [0.08-1.21]	0.093	-	-
**IVIG** (ref=No)	20.57 [2.41-175.43]	0.006	50.95 [2.25-115.19]	0.014
**PLEX** (ref=No)	0.36 [0.10-1.36]	0.132	-	-
